# Squamoid Eccrine Ductal Carcinoma

**Published:** 2015-06-22

**Authors:** Kelly Segars, Jared M. Gopman, Joshua B. Elston, William Carter, Michael A. Harrington

**Affiliations:** ^a^Nova Southeastern University College of Osteopathic Medicine, Davie, Fl; ^b^Division of Plastic Surgery, Department of Surgery, University of South Florida Morsani College of Medicine, Tampa; ^c^Division of Plastic Surgery, James A. Haley Veterans Affairs Hospital, Tampa, Fl

**Keywords:** eccrine carcinoma, skin cancer, squamoid ductal, perineural invasion, local recurrence

## DESCRIPTION

An 89-year-old man presented with a rapidly progressing lesion of the right lower back initially diagnosed as squamous cell carcinoma (SCC). Excisional biopsy showed primary squamoid eccrine ductal carcinoma (SEDC) with indistinct borders involving the deep margin. Further excision with 1-cm margins demonstrated perineural invasion. Oncology was consulted and recommended observation.

## QUESTIONS

1. **What is SEDC?**
2. **How does SEDC present?**
3. **What are some differential diagnoses of SEDC?**
4. **What are the current treatment and staging recommendations?**

## DISCUSSION

The group of eccrine carcinomas (ECs) in its entirety comprises less than 0.01% of all cutaneous malignancies.^1^^-^3 SEDC is a rare cutaneous malignancy that originates from eccrine glands and exhibits both squamous and adnexal ductal characteristics.^1^ It is the rarest variant of which only 10 to 15 reported cases exist, mostly in the dermatology literature. SEDC can arise de novo either from normal adnexal tissue or from malignant transformation of a preexisting benign eccrine tumor. Like other ECs, SEDC has a strong predilection for local recurrence as well as a high metastatic potential reportedly up to 50%.^3^

SEDC typically presents in patients older than 50 years with a variable appearance on the head, neck, and trunk. It can present as a slow or rapidly growing nodule or plaque, exophytic and locally destructive, or entirely benign-appearing.^1^^,^^2^^,^^4^ Extensive sunlight exposure and immunosuppression are risk factors reported to increase the risk of all ECs.^4^ Histologically, SEDC is noted to be locally aggressive and frequently courses along perineural tissue.^2^^,^^5^ This propensity to spread along nerves likely contributes to the high local recurrence and distant spread.

The differential diagnosis for SEDC includes SCC, other ECs (porocarcinoma, microcystic adnexal carcinoma), metastatic disease, Merkel cell carcinoma, and benign neoplasms.^1^^,^^2^^,^^4^^,^^5^ In this case, the initial histological diagnosis was SCC in the background of a scar. SEDC is commonly misdiagnosed for SCC; however, the distinction is essential, as recurrence and metastasis rates are far greater.^2^ The pathological diagnosis of SEDC is difficult; however, experienced dermatopathologists have shown that specific immunological staining can be helpful in differentiating a primary versus metastatic process as well as other similar appearing carcinomas.^1^^-^3^,^^5^

Because of the poorly understood malignant potential, difficulty of diagnosis, and the rarity of these tumors, there are no widely accepted treatment guidelines. Surgical excision appears to be the primary curative modality, with little data on the benefit of adjuvant therapy.^4^ Reports of local recurrence rates after surgical excision are reportedly up to 70%.^3^^,^^5^ Current literature reports successful use of micrographic excision, with 0% to 5% recurrence rates after an average 30.9 months of follow-up.^6^^,^^7^ Another series reported no recurrences in 19 patients with a variety of ECs treated with micrographic excision and 4- to 5-mm margins at a follow-up period of 29 months. Radiation and general oncology consultations should be considered in deciding whether radiation therapy is indicated for positive margins with perineural invasion or those presenting with metastatic disease. It has been proposed that FFDG PET/CT (positron emission tomography with 2-deoxy-2-[fluorine-18]fluoro-D-glucose integrated with computed tomography) imaging is indicated to stage patients with a diagnosis of SEDC, based on the high likelihood for metastatic disease.^8^ The evidence for the role of sentinel lymph node biopsy with SEDC is lacking; however, the senior author has performed this procedure at the recommendation of an interdisciplinary tumor board conference. It stands to reason that a locally aggressive disease with high metastatic capabilities could potentially benefit from more aggressive surgical margins (1 cm) to disrupt local lymphatic channels and possibly decrease the risk for local recurrence.

SEDC is a rare variant cutaneous adnexal tumor derived from the family of ECs with high metastatic potential and a sometimes misleadingly unimpressive presentation. Diagnosis can be difficult and requires an experienced dermatopathologist. While no formal treatment or staging recommendations exist, micrographic excision has met with favorable results versus formal excision with 4- to 5-mm margins.^5^ The need for appropriate excision with clear margins is critical, given the propensity to course along nerves, recur locally, and spread to distant sites if left untreated.

## Figures and Tables

**Figure 1:**
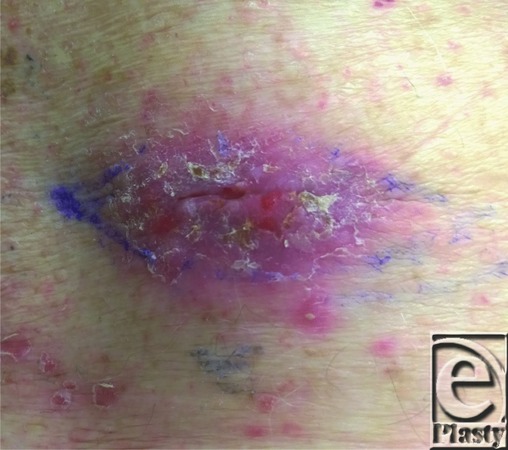
Right lower back lesion diagnosed as squamous cell carcinoma on previous shave biopsy with final excisional biopsy results of squamoid ductal eccrine carcinoma.
